# Efficacy of Sirolimus in Patients Requiring Tracheostomy for Life-Threatening Lymphatic Malformation of the Head and Neck: A Report From the European Reference Network

**DOI:** 10.3389/fped.2021.697960

**Published:** 2021-09-30

**Authors:** Annegret Holm, Maroeska te Loo, Leo Schultze Kool, Päivi Salminen, Veronica Celis, Eulalia Baselga, Sophie Duignan, Veronika Dvorakova, Alan D. Irvine, Laurence M. Boon, Miikka Vikkula, Nader Ghaffarpour, Charlotte M. Niemeyer, Jochen Rössler, Friedrich G. Kapp

**Affiliations:** ^1^Division of Pediatric Hematology and Oncology, Department of Pediatrics and Adolescent Medicine, Medical Center – University of Freiburg, Faculty of Medicine, University of Freiburg, Freiburg, Germany; ^2^VASCERN VASCA European Reference Centre, Paris, France; ^3^Radboud University Medical Centre, Nijmegen, Netherlands; ^4^Department of Pediatric Surgery, Children's Hospital, Helsinki University Hospital and University of Helsinki, Helsinki, Finland; ^5^Sant Joan de Déu Hospital, Barcelona, Spain; ^6^Paediatric Dermatology, Our Lady's Children's Hospital Crumlin, Dublin, Ireland; ^7^National Children's Research Centre, Dublin, Ireland; ^8^Clinical Medicine, Trinity College Dublin, Dublin, Ireland; ^9^Center for Vascular Anomalies, Division of Plastic Surgery, Saint-Luc University Hospital, Brussels, Belgium; ^10^Human Molecular Genetics, de Duve Institute, University of Louvain, Brussels, Belgium; ^11^Department of Pediatric Surgery, Karolinska University Hospital, Stockholm, Sweden; ^12^Division of Pediatric Hematology and Oncology, Department of Pediatrics, Inselspital, Bern University, Hospital, University of Bern, Bern, Switzerland

**Keywords:** sirolimus, rapamycin, lymphatic malformation, vascular anomaly, tracheostomy, tracheostoma

## Abstract

Extensive lymphatic malformations (LMs) of the head and neck region may require tracheostomy to secure the airway. Treatment of these life-threatening LMs is usually multimodal and includes sclerotherapy and surgery, among others. Recently, systemic therapy with sirolimus has been introduced as an effective treatment for venous and lymphatic malformations; its efficacy and safety profile in patients with extensive LM requiring tracheostomy are, however, as yet not fully known. We performed a retrospective, multicenter review and identified 13 patients with an extensive LM of the head and neck region, who previously underwent placement of tracheostomy and subsequently received sirolimus treatment with the aim to improve the local respiratory situation and remove the tracheostomy. Under sirolimus therapy, tracheostomy could be reversed in 8/13 (62%) patients, a further 2/13 (15%) patients improved markedly, and removal of the tracheostomy was planned at the time of writing, while 3/13 (23%) patients showed insufficient or absent response to sirolimus, rendering tracheostomy reversal not feasible. The median duration of sirolimus treatment until removal of tracheostomy was 18 months (range, 8 months to 5.6 years). Adverse events of sirolimus therapy were common [10/13 (77%) patients], yet the majority of these were mild [9/10 (90%) patients] and only one severe adverse event was recorded, with ulceration and necrosis at a catheter insertion site. In conclusion, sirolimus can be considered an effective and safe salvage treatment in patients with extensive LM even after placement of a tracheostomy, as closure of the latter was possible in the majority of patients (62%) of our retrospective cohort. A better understanding of when to start sirolimus therapy, of the duration of treatment, and of factors allowing the prediction of treatment response will require further investigation.

## Introduction

Extensive lymphatic malformations (LMs) of the head and neck region can be life-threatening due to infiltration of vital structures of the airway and may require early intubation, e.g., by *ex utero* intrapartum treatment (EXIT) procedure, mechanical ventilation, and even long-term placement of tracheostomy to secure the airway and/or feeding tubes to realize nutrition. The majority of extensive cervical LMs are diagnosed pre- or post-natally and generally require treatment early during the neonatal period. Treatment modalities depend on the extent of macrocystic, microcystic, or mixed components of the LM and include multimodal approaches such as repetitive sclerotherapy, surgical resection, and laser therapy with often unsatisfying results. The complexity and the variable presentation of extensive head and neck LM complicate the understanding of the natural history and the response to different treatment modalities of this rare disease. Several systems for staging and outcome measures have been proposed (1, 2). Sirolimus has recently been introduced as an alternative systemic treatment option in complex vascular anomalies (3, 4) and it has been further demonstrated that neonates with extensive LM benefit from low-dose systemic treatment with sirolimus immediately after birth, while experiencing only mild side effects (5).

Sirolimus is an mTOR inhibitor and blocks downstream signaling of the PI3K/AKT/mTOR pathway that governs physiological vascular development and angiogenesis (6, 7). In pediatric patients, experience with sirolimus treatment as an immunosuppressant agent is scarce and it has been gained mainly from kidney transplantation patients (8). In the context of vascular anomalies, and specifically in pediatric patients, the efficacy and safety of sirolimus, however, is still a matter of ongoing research. Published data are promising (3–5, 9–11), yet the drug still lacks regulatory approval for the treatment of vascular anomalies (3, 5).

In this multicenter retrospective study, we analyzed our experience with 13 patients treated with sirolimus in multidisciplinary vascular anomaly centers of the VASCA working group of the European Reference Network VASCERN after insufficient standard therapies. All patients required tracheostomy due to a life-threatening extent of an LM of the head and neck region with compression of the airway. The study focused on efficacy with respect to successful reversal of tracheostomy and safety of the drug.

## Materials

We performed a retrospective multicenter chart review of 13 patients with extensive LM of the head and neck region that had previously undergone placement of a tracheostomy at a median age of 1 month (range, 1 day to 45 months). Patients suffered from an isolated LM of the head and neck region and were not subjected to an underlying complex lymphatic anomaly. All patients were treated with sirolimus between January 2013 and December 2020 (treatment is still ongoing in nine patients) with the aim to remove the tracheostomy. We assessed the efficacy, which was defined as achieving removal of the tracheostomy, and the safety of sirolimus, defined as the occurrence of adverse events (AEs). Patients and their guardians were informed of the risks, benefits, and therapeutic alternatives before treatment with off-label sirolimus. This study is covered by the ethical committee number Freiburg 464/19.

### Patient Data

Analyzed patient variables included basic patient characteristics, location and extent of the LM, symptoms, other previous and concomitant treatment modalities and their effects, treatment with sirolimus, dosage and trough level targets as well as treatment duration and response, and AEs and severe adverse events (SAEs). The definition of AEs was according to Good Clinical Practice (GCP) rules and included any untoward medical occurrence, with SAE including events that resulted in death, were life-threatening, required inpatient hospitalization or caused prolongation of existing hospitalization, resulted in persistent or significant disability/incapacity, might have caused a congenital anomaly/birth defect, or required intervention to prevent permanent impairment or damage. We used the following grading system: grades 1 and 2 represent non-SAEs, and grades 3–5 represent SAEs: 3 for severe or medically significant but not immediately life-threatening, 4 for life-threatening, and 5 for death.

### Treatment

Treatment with sirolimus was proposed to promote reversal of tracheostomy in patients with extensive LM of the head and neck region after results of other treatment modalities were not satisfactory. Treatment with sirolimus resulted in reversal of tracheostomy after a median of 18 months (range, 8 months to 5.6 years) of treatment initiation. The median age at tracheostomy removal was 2 years (range, 11 months to 6 years) and the administered dose of sirolimus was adapted according to target trough levels (5–15 ng/ml), respectively. Efficacy criteria included clinical improvement of symptoms and removal of tracheostomy.

### Data Analysis

A retrospective data analysis of 13 patients treated at European Reference Centers was performed. Data are presented in percentage from total and medians according to the respective ranks.

## Results

### Patient Characteristics

Tracheostomy had been performed in 13 patients with life-threatening LM of the head and neck region prior to treatment with sirolimus. One patient (P07) had additionally received a gastrostomy simultaneously with the placement of the tracheostomy at the age of 5 months to facilitate feeding. Six (46%) patients were female, and seven (54%) were male. The LM was diagnosed prenatally in four (31%) patients, at birth in eight (62%) patients, and at 1 month of age in 1 (8%) patient. All patients had acute or sub-acute obstruction of the upper airway caused by the LM, which also involved the tongue and larynx in six patients (46%) and the mediastinum in two patients (15%). Prior to treatment with sirolimus, 10 (77%) patients received sclerotherapy with OK432, doxycycline, or bleomycin; 6 (46%) received surgical resection; and 5 (38%) received laser therapy (Tables 1, 2).

**Table 1:** Summary of clinical patient characteristics and treatment other than and prior to sirolimus (order according to treatment response).

				**Treatment modalities**			
**Patient ID**	**Gender**	**Age at onset**	**Localization and extent**	**Sclerotherapy**	**Laser therapy**	**Resection**	**Other**
P01	F	At birth	Tongue, floor of mouth, mandibular, submentally up to left ear, neck				RFA
P02	M	At birth	Right cervical region extending intrathoracically				
P03	M	Intrauterine	Face, neck, mediastinum extending to carina				
P04	M	Intrauterine	Right side of the face and neck, right parapharyngeal involvement				
P05	F	1 m	Base of mouth, oropharynx, anterior cervical region, both parapharyngeal spaces and left temporal fossa				
P06	F	At birth	Tongue				
P07	M	At birth	Extensive cervical and facial region, tongue				
P08	M	At birth	Lung, pleura with pleural and pericardial effusions, right parotid, subglottis, subcutaneous, splenic and mesenteric lesions				Pericardio-centesis
P09	F	At birth	Tongue, floor of mouth, partial trachea obstruction, face, neck				
P10	M	Intrauterine	Floor of mouth, larynx, neck				
P11	F	At birth	Extensive neck, laryngeal cleft with compression of trachea				
P12	F	At birth	Tongue, floor of mouth, mandibular, cheeks				
P13	M	Intrauterine	Tongue, floor of mouth, mandibular, neck and larynx				

*F, female, M, male, m, month, RFA, radiofrequency ablation*.

**Table 2:** Summary of tracheostomy and treatment with sirolimus (order according to treatment response) and associated adverse events.

**Patient ID**	**Age at placement of tracheostoma**	**Age at start of sirolimus**	**Trough levels of sirolimus [ng/mL]**	**AE/SAE under sirolimus**	**Duration of sirolimus therapy while under tracheostomy [months]**	**Status of tracheostomy, at age of**	**Others**
01	5 m	8 y	3–6	–	29, discontinued at age 13 y, 9 m	Removal, 10 y, 5 m	Severe cervical swellung under parotitis epidemica tracheostomy from ages 1 m to 2 y, and 5 y to 10 y, 10 m sirolimus discontinued after tracheostomy reversal, restarted due to increase of LM PIK3CA mutation, switch to alpelisib at age 17 y, 6 m ongoing monthly avastin since age 4 y
02	9 m	10 m	5–10	Mild infections	16, Still ongoing	Removal, 2 y, 2 m	
03	2 d	2 m	2–5	Ulceration and necrosis at pigtail catheter site, trachea reconstruction with cartilage graft	20, Still ongoing	removal, 2 y, 10 m	
04	1 m; 5 y	9 y	5–10	Lymphopenia	12, Still ongoing	Removal, 10 y, 10 m	
05	1 m	3 y, 1 m	4–5	Mild infections	10, Still ongoing	Removal, 3 y, 11 m	
06	6 w	15 m	3.5–5.6	–	21, Still ongoing	Removal, 3 y	
07	5 m	11 y, 11 m	5–10	Skin toxicity °I/II, mild infections	67, Discontinued at 17 y, 6 m	removal, 17 y, 6 m	
08	3 y, 9 m	3 y	3–5	Lymphopenia	8, discontinued at 6 y, 11 m	Removal, 5 y, 2 m	
09	14 d	1 m	3.8–4.7	Mild infections	32, Still ongoing	Removal planned	
10	1 d	2 m	5–10	Mild infections	31, Still ongoing	Removal planned	
11	2 m	3 y, 9 m	3.3–4.6	Hypophosphatemia, mild infections	23, Still ongoing	Still required	
12	2 d	18 y	10–15	Recurrent FUO	7	Still required	
13	2 d	5 y	5–10	–	5	Still required	

*m, months; y, years; FUO, fever of unknown origin; PIK3CA, Phosphatidylinositol-4,5-Bisphosphate 3-Kinase Catalytic Subunit Alpha*.

### Tracheostomy and Treatment With Sirolimus

**Figure 1:**
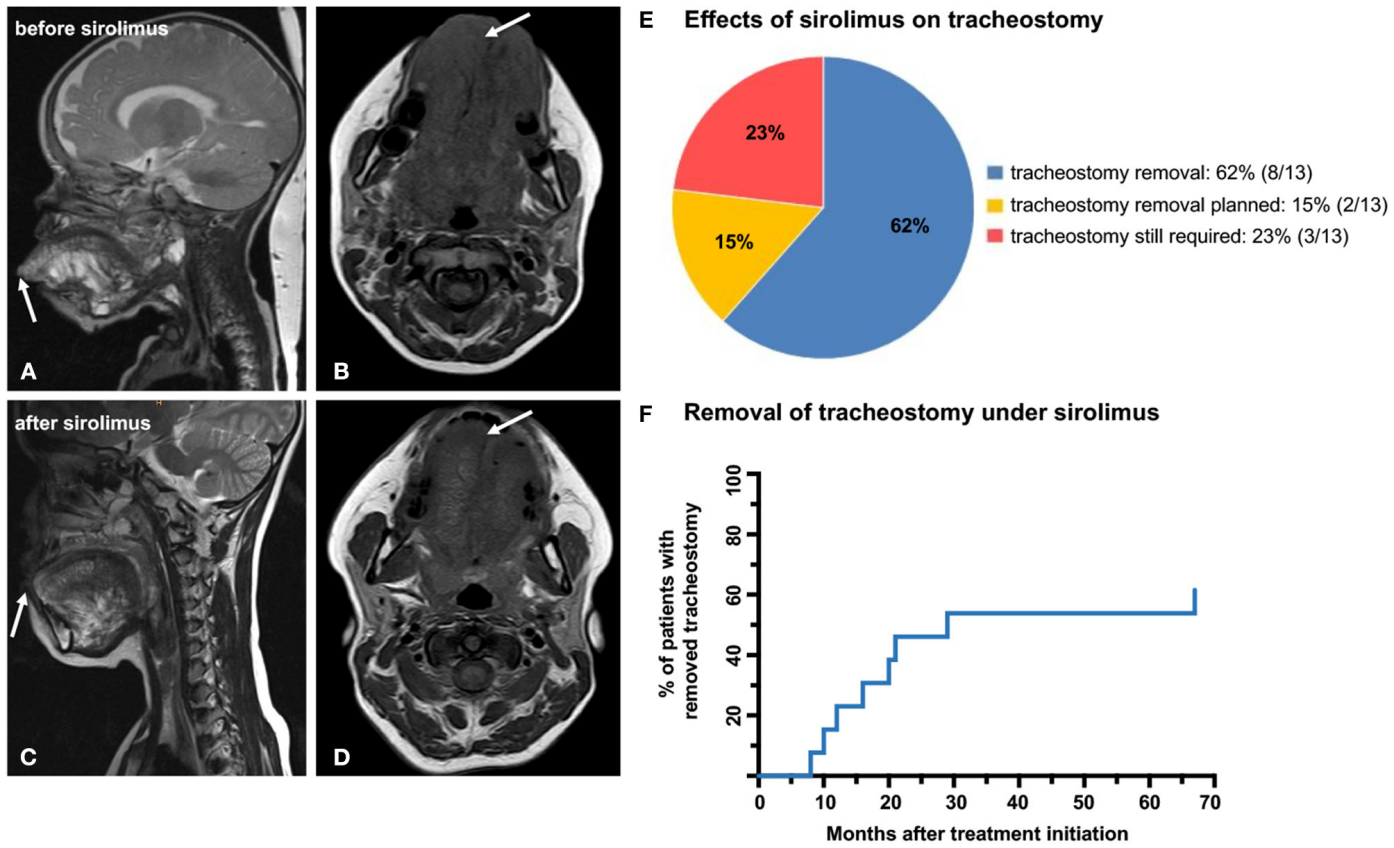
MRI images of P01 before **(A,B)** and after **(C,D)** treatment with sirolimus shows marked size reduction of the LM affecting the tongue and the floor of the mouth (arrows). As a result of the sirolimus therapy, the patient was able to completely close her mouth prior to reversal of the tracheostomy **(C,D)**. **(E)** Pie chart depicting the distribution of patients: in 8/13 patients, sirolimus showed complete response and the tracheostomy could be reversed; 2/13 patients showed good response with planned removal of the tracheostoma; and in 3/13 patients, the tracheostomy could not be removed due to insufficient response. **(F)** Kaplan–Meier curve illustrating the percentage of patients (62%), in whom tracheostomy could be removed in months after treatment initiation with sirolimus.

Tracheostomy was performed at a median age of 1 month (range, 1 day to 45 months). In two patients (P05 and P13), an EXIT procedure was performed, while the other patients were born by elective cesarean section or per natural route and successfully stabilized during the neonatal first aid. Dosing of sirolimus depended on age at treatment initiation, which varied widely with a median of 37 months (range, 1 month to 18 years). Three patients (P05, P10, and P13) were started early on sirolimus at 1 (P10) and 2 months (P05 and P13). P05 started with low-dose sirolimus including a 50% reduction of the recommended dose of 0.8 mg/m^2^/12 h (3, 5). All other patients received the recommended dose of 0.8 mg/m^2^/12 h. Following initiation of sirolimus therapy, reversal of tracheostomy was accomplished in eight patients (62%, P1–P8) after a median of 18 months (range, 8 months to 5.6 years) of treatment with sirolimus. The median age at tracheostomy reversal was 2 years (range, 11 months to 6 years). In another two responding patients (P09 and P10), removal of tracheostoma was planned at the time of writing. One patient (P11) experienced clinical stabilization with decrease in pain and infections, but without relevant size reduction of the LM, so that removal of the tracheostomy was not an option. In two patients (P12 and P13), sirolimus was discontinued early after 5 and 7 months of therapy for lack of clinical benefit (Figure 1, Table 2).

**Figure 2:**
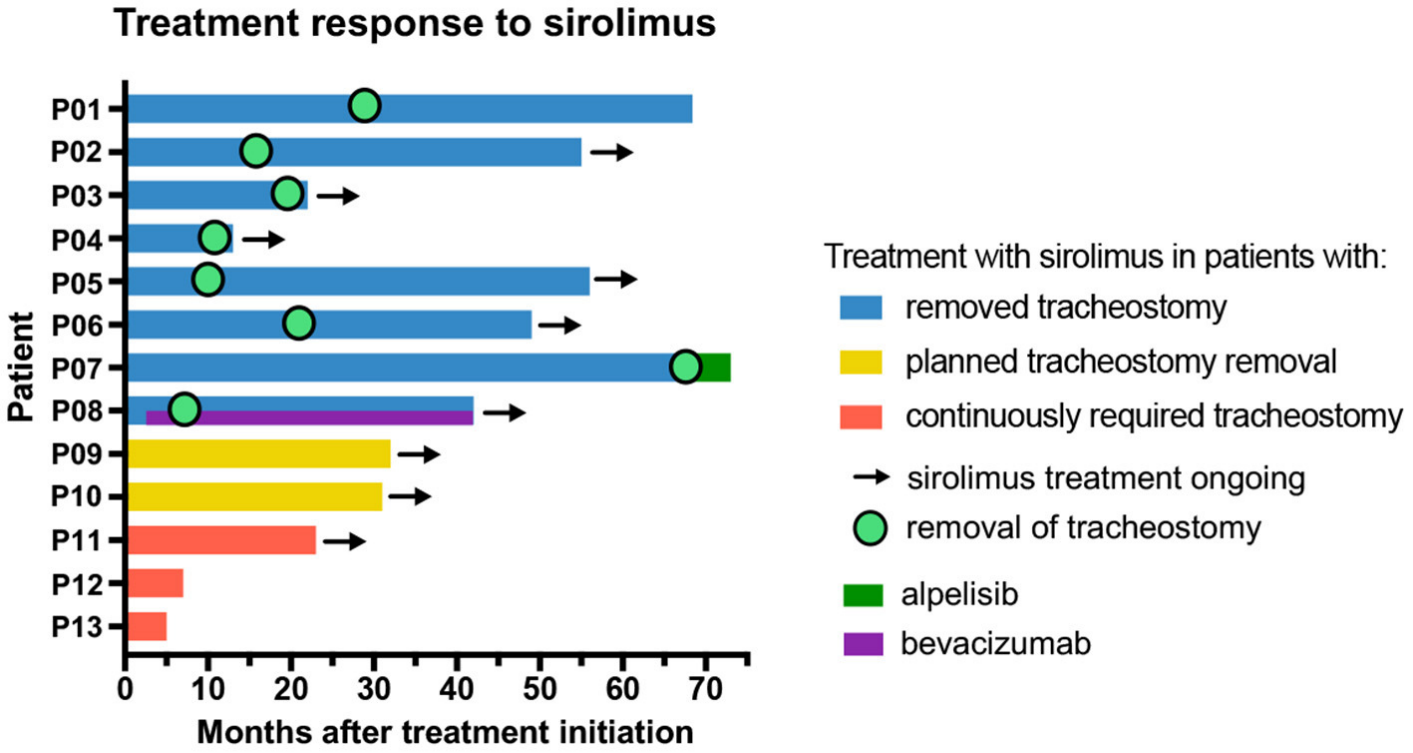
Overview of patient groups differentiated by treatment response (blue), good response (yellow), and insufficient treatment response (P11, P12, and P13) (red). The graph further illustrates total treatment duration with sirolimus, time to removal of tracheostomy after sirolimus treatment initiation, as well as (simultaneous) treatment with the PI3K inhibitor alpelisib and the VEGFA inhibitor bevacizumab (in months).

The clinical benefit of sirolimus therapy was not restricted to the removal of the tracheostomy. For instance, treatment response in P01 included a significant decrease in the size of the tongue that enabled the patient to regain the ability to close her mouth completely (MRI images Figures 1A–D, 2). In 9/13 (69%) patients (P02–P06 and P08–P11), treatment with sirolimus is ongoing, with intermittent but unsuccessful attempts to taper and discontinue the drug. Additional medical treatments were performed in two patients. P07 was found to harbor a somatic *PIK3CA* mutation and was started on alpelisib—an inhibitor of the phosphatidylinositol-4,5-bisphosphate 3-kinase, catalytic subunit alpha—with the aim to sustain a stable clinical condition following removal of tracheostomy. Sirolimus was discontinued in this patient after a total of 67 months of treatment initiation simultaneously with the introduction of alpelisib and he remained in a stable respiratory condition thereafter. P08 had an incomplete response to 8 months of sirolimus therapy; bevacizumab—an inhibitor of the vascular endothelial growth factor A (VEGFA)—was added and the tracheostomy could be removed after 5 months of simultaneous treatment. Both sirolimus and bevacizumab are still ongoing in this patient for clinical stabilization (Figures 1, 2).

Treatment with sirolimus is ongoing in a total of 9/13 (69%) patients (P02–P06 and P08–P11). Of these nine patients, treatment was continued in six patients after successful removal of tracheostomy (P02–P06 and P08). Two patients (P09 and P10) showed size reduction of the LM and clinical stabilization. In P09, sirolimus was intermittently discontinued due to relapsing mild infections resulting in recurrent size increase of the LM. P10 showed a treatment response with adequate size reduction, cessation of bleeding and inflammation resulting in respiratory stabilization. As mentioned above, reversal of the tracheostomy is now anticipated in both patients. P11 experienced clinical improvement without size reduction under sirolimus but the response was insufficient for reversal of the tracheostomy (Table 2).

AE under treatment with sirolimus occurred in 10/13 (77%) patients. They included mild viral infections in 7/10 (70%) patients (P02, P03, P05, P07, and P09–P11), lymphopenia in 2/10 (20%) patients (P04 and P08), and low-grade skin toxicity, hypophosphatemia, as well as recurrent mild fever of unknown origin in 1/10 (10%) patients each (P07, P11, and P12). There was one SAE with ulceration and necrosis at a pigtail catheter insertion site necessitating hospitalization and discontinuation of sirolimus therapy (P03). In addition, the same patient suffered from inflammation and scarring at the tracheostoma that required repetitive excisions of a substomal intratracheal lump 15 and 28 months after sirolimus treatment initiation and eventually a reconstruction of the trachea with a cartilage graft.

## Discussion

To the best of our knowledge, this is the first study to assess the efficacy of sirolimus in patients requiring tracheostomy for life-threatening LM of the head and neck region. The main findings of this study are that (i) sirolimus is an effective salvage treatment in patients with an extensive LM of the head and neck region requiring tracheostomy, (ii) tracheostomy may be removed in a majority of patients under sirolimus therapy, and (iii) AEs under sirolimus therapy are common, yet well-controllable if monitored carefully.

In patients undergoing tracheostomy due to acute compression of vital structures by extensive LM of the head and neck region, treatment with sirolimus proved to be an effective salvage option with reversal of tracheostomy in a significant number of patients (8/13, 62%) with an additional 2/13 (15%) patients planned for tracheostomy reversal. One of these patients (P07) received alpelisib following removal of tracheostomy after long-term treatment with sirolimus and its discontinuation after 5.6 years. While preliminary data are promising in patients with PROS diseases (12), the role of alpelisib in the treatment of head and neck LM remains to be defined. Another patient (P08) received bevacizumab in addition to sirolimus, and both drugs are still ongoing to stabilize the patient's respiratory condition. Bevacizumab is a monoclonal antibody and, besides its well-known inhibitory effect on tumor angiogenesis, attenuates VEGFA-mediated signaling in complicated lymphatic anomalies (CLAs) (13, 14). In our cohort, treatment with sirolimus resulted in relevant clinical and radiological improvement of the LM, as was expected from the literature. Optimal dosing and (serum) target trough levels considering individual patient characteristics have been evaluated recently but are still a matter of an ongoing debate (3, 5). According to our data, we did not see a correlation between dosage, target trough levels (2–15 mg/ml), initial size of the lesion, gender, or AE and treatment response in our relatively small patient cohort. In previous publications, trough levels of 10–15 ng/ml were recommended (3, 5). In fact, growing experience with sirolimus therapy in the field of vascular anomalies in recent years has demonstrated that treatment response does not improve with increased sirolimus trough levels (15). Interestingly, the median age at treatment initiation of sirolimus with 37 months in all patients and 26 months in successfully treated patients (P01–P10) was younger than in those with insufficient treatment responses (P11–P13) with 60 months (Tables 1, 2). Even though these data are not significant, they suggest that treatment initiation of sirolimus at a young age might be beneficial with regard to treatment response rates [10/13 (77%) responders vs. 3/13 (23%) non-responders]. The safety of early treatment initiation has previously been shown in a small cohort of neonates (5).

While 3/8 patients (38%) achieved removal of tracheostomy within 1 year, this took longer in the remaining patients (up to 67 months). We propose that this may be due to the gradual reduction in treatment response as well as the need for a stable clinical status over a longer time period before tracheostomy removal. It is important to communicate a possibly long treatment duration with patients and their families.

AEs were common but almost exclusively non-severe and within the spectrum of expected side effects of sirolimus. We would attribute the only SAE in our cohort (necrosis at a pigtail catheter insertion site and inflammation and scarring at the tracheostoma) to an expectedly impaired wound healing under sirolimus treatment. The safety profile of sirolimus in the context of vascular anomalies (9–11) is similar to what has been demonstrated earlier in pediatric kidney transplantation patients (8, 16), with SAEs being rarely reported especially in vascular anomalies (9). However, the exact spectrum of side effects of sirolimus in this special patient population is, as yet, not fully known and needs further investigation. Other considerations, e.g., hypothetical long-term effects of immunosuppression with sirolimus on a developing immune system of an infant, should also be assessed further in the future.

Most viral infections under sirolimus involved the respiratory tract, as would be expected in a pediatric patient cohort. Furthermore, LM of the head and neck region may additionally compress relevant anatomic structures of the airway, thereby further predisposing to lung infections. Along these lines, vaccines in patients with complex vascular anomalies treated with sirolimus and therefore susceptible to infections are to be critically reconsidered (9). Whenever possible, vaccines should be completed before treatment initiation with sirolimus. As this might not be feasible in all patients (e.g., neonates), inactivated vaccines may be administered during therapy, despite the fact that the immunogenic response might not be optimal. Although we generally recommend delaying immunization with attenuated life vaccines until the patient is off sirolimus therapy, timing may not be feasible in patients who require lengthy sirolimus therapy for clinical stabilization as was the case in four of our patients in this study. To illustrate, P02 had not received attenuated vaccines as sirolimus was started at the age of 10 months. He developed severe epidemic parotitis after 3 years and 9 months of treatment with sirolimus at the age of 4 years and 7 months. Due to extensive cervical swelling and a secondary inflammatory response of the LM, the patient came close to necessitating mechanical ventilation again. This exemplifies that individual consideration of the risks and benefits of inactivated and attenuated vaccines in these vulnerable patients is critical.

A weakness of our study is its retrospective nature and the lack of a control group. Extensive cervical LM with acute or subacute airway obstructing are extremely rare and life-threatening disease states requiring prompt and often multimodal treatment. Alternative methods to collect data on this highly selected patient cohort were not feasible under these conditions. With the abovementioned limitations in mind, it is difficult to assess the natural history as well as the value of selected treatment modalities in patients with life-threatening head and neck LM. On the basis of our data, we hypothesize that sirolimus may have the potential to significantly contribute to the improvement of our patient's life-threatening condition. We are aware that the efficacy of sirolimus in our patients may be at least in part confounded by the multimodal treatments prior to and during sirolimus therapy and that the naturally increasing size of the airway may have further impacted tracheostomy removal, as has been reported for other indications of tracheostomy in pediatric patients (17). However, the rate of successful tracheostomy removal in our patient cohort treated with sirolimus is encouraging and we expect that multimodal treatment approaches including sirolimus, sclerotherapy, and surgery at interdisciplinary vascular anomaly centers will increasingly be applied in the future and may contribute to an improved prognosis of severely affected patients. To refine the treatment with sirolimus, many open questions remain to be answered such as dosage, treatment duration, and tapering of sirolimus. Yet, this should not discourage physicians from treating severely affected patients with sirolimus, even after extensive pre-treatment. Sirolimus is still considered an “off-label” therapy in complex vascular anomalies. We hope that our study will pave the way for future international studies conducted in a well-planned manner, for instance, by comparing two drugs inhibiting the PIK3CA/mTOR pathway, e.g., sirolimus and alpelisib, to further delineate the value of these drugs in the treatment of extensive LM of the head and neck region.

## Conclusions

Sirolimus is an “off-label” treatment that can be considered an efficient and safe salvage treatment option in patients with complex, life-threatening and treatment-resistant LM of the head and neck requiring tracheostomy, as it significantly (62% in our series) reduces the need for tracheostomy following size reduction of LM and respiratory stabilization. AEs under sirolimus were common (77%), but mostly remained mild and controllable if monitored carefully. Adequate selection of patients and timing of treatment with sirolimus require further investigation.

## Data Availability Statement

The original contributions presented in the study are included in the article/Supplementary Material, further inquiries can be directed to the corresponding author/s.

## Ethics Statement

The studies involving human participants were reviewed and approved by Ethical Committee number 464/19, University Hospital Freiburg. Written informed consent to participate in this retrospective study was provided by the participants' legal guardian/next of kin. Written informed consent was obtained from the minor(s)' legal guardian/next of kin for the publication of any potentially identifiable images or data included in this article.

## Author Contributions

AH, JR, and FK: conceptualization and writing—original draft preparation. JR and FK: conceptualization and supervision. MtL, LS, PS, VC, EB, SD, VD, AI, LB, MV, NG, and CN: writing—review and editing. All authors patient contribution.

## Funding

This work was supported in part by the Center for Vascular Anomalies, Freiburg Center for Rare Diseases. FK was supported by an EXCEL-Fellowship of the Faculty of Medicine, University of Freiburg, funded by the Else–Kröner–Fresenius–Stiftung. The article processing charge was funded by the Baden-Wuerttemberg Ministry of Science, Research and Art and the University of Freiburg in the funding programme Open Access Publishing.

## Conflict of Interest

LB receives fees from Pierre Fabre, outside the submitted work. CN receives consulting fees from Novartis and BMS outside the submitted work. JR reports personal fees from SOBI, Roche, and Bayer for Advisory Board membership as well as consulting fees from Pierre Fabre, all outside the submitted work. FK reports consulting fees from Novartis, outside the submitted work. The remaining authors declare that the research was conducted in the absence of any commercial or financial relationships that could be construed as a potential conflict of interest.

## Publisher's Note

All claims expressed in this article are solely those of the authors and do not necessarily represent those of their affiliated organizations, or those of the publisher, the editors and the reviewers. Any product that may be evaluated in this article, or claim that may be made by its manufacturer, is not guaranteed or endorsed by the publisher.

## Abbreviations

AE: Adverse event
AKT: Protein kinase B
EU: European Union
LM: Lymphatic malformation
ICU: Intensive care unit
MRI: Magnetic resonance tomography
mTOR: Mechanistic target of rapamycin
OK432: Picibanil
PI3K: Phosphatidylinositol 3-kinase inhibitor
PIK3CA, Phosphatidylinositol-4: 5-bisphosphate 3-kinase catalytic subunit alpha
PROS: PIK3CA-related overgrowth syndrome
SAE: Severe adverse event
VA: Vascular anomaly
VASCERN: European Reference Network on Rare Multisystemic Vascular Diseases
VEGFA: Vascular endothelial growth factor A.
